# Modified Micro-Mechanics Based Multiscale Model for Damage Analysis of Open-Hole Composite Laminates under Compression

**DOI:** 10.3390/ma15155105

**Published:** 2022-07-22

**Authors:** Meng Wang, Xiaochen Hang

**Affiliations:** 1 Jiangsu Key Laboratory of Environmental Impact and Structural Safety in Engineering, School of Mechanics & Civil Engineering, China University of Mining and Technology, Xuzhou 221116, China; 2 College of Mechanical and Electronic Engineering, Nanjing Forestry University, Nanjing 210037, China; xchang@njfu.edu.cn

**Keywords:** micro-mechanics failure theory, open-hole laminates, interface, clustering method, feed-forward neural network

## Abstract

The multiscale model based on micro-mechanics failure theory is modified to consider complex internal structures, including a fiber random arrangement pattern and interface with the clustering method. Then, a feed-forward-neural-network (FFNN)-based damage evolution method is developed to evaluate the macroscale property degradation. The progressive damage analysis of open-hole laminates under compression is conducted to validate the modified multiscale method. The predicted results reveal that the interface results in the premature initiation of damage, and the fiber random arrangement pattern contributes to the decrease in the predicted compression responses. The developed FFNN-based method aimed at degradation results in an increase in the predicted compression strength. For the fiber random distribution pattern, the increase in percentage of predicted compressive strength is 6.0%, which is much larger than the value for the fiber diamond distribution pattern.

## 1. Introduction

Composites have been widely used in aerospace engineering, automotive engineering, civil engineering and so on. Inevitably, holes in the laminates would be drilled for connection. Thus, the prediction of strength values and revelation of failure mechanisms for the open-hole laminates become critical steps for the strength verification of large composite structures.

The experimental methods have been used for revealing the failure mechanisms [1,2]. However, due to the limitation of the experimental technique, the damage process at the macroscale and microscale cannot be obtained simultaneously. Different kinds of numerical methods have been developed for the progressive damage analysis of laminates considering the hole. Two critical parts should be included in the analysis: one is the failure criteria for the damage initiation judgment and another one is the damage evolution method. The Tsai–Wu criterion and Hoffman criterion were compared by Zhou et al. [3] to evaluate the effects of failure criteria on the predicted compressive behaviors of open-hole laminates. The Hashin failure criteria were adopted by Shimizu et al. [4] for the damage initiation judgment of laminates in the longitudinal and transverse direction. Zhang et al. [5] modified the failure criteria with considering fiber kinking and shear non-linearity. After one certain failure mode is activated, the damage evolution method needs to be adopted to degrade the elastic parameters of laminates. The damage evolution models can be divided into two groups, including sudden degradation models and gradual degradation models [6]. For the sudden degradation models, the material properties are reduced instantaneously to some fraction of the original properties once a failure mode is activated [7,8]. For the gradual degradation models, the damage parameters for a failure mode increase from zero to one gradually based on empirical functions or a physical failure model [9,10,11]. However, with these damage initiation and evolution models, only the progressive damage process can be obtained, but that at the microscale cannot be revealed.

The micro-mechanics-failure-theory (MMF)-based multiscale method has also been widely used for the progressive damage analysis of open-hole laminates [12,13,14,15,16]. Within this multiscale framework, the elastic analysis of the microscale representative volume element (RVE) is conducted first to obtain the amplification factors for the critical points of the RVE. Then, the amplification factors are extracted and used to calculate the stress/strain values at the microscale through the macroscale stress/strain values. With these stress/strain values at the microscale, the damage initiation in the constituents can be judged. The calculation process based on the amplification factors is named as the localization process, with the information transferred from the macroscale model to the microscale model [17]. At last, damage evolution models are used to degrade the elastic parameters of macroscale models, and this calculation process is named as the homogenization process [17].

For the localization process, in the original MMF-based multiscale framework, only fiber regular distribution microscale models were considered, and some regular reference points from the microscale RVE were chosen for the extraction of amplification factors [12,14]. To have the fiber random distribution patterns considered, the clustering method was adopted to modify the extraction process of amplification factors so that the effects of the fiber random distribution patterns on the damage initiation at the microscale can be revealed [18]. The effects of the interface debonding between the fiber and matrix on the behavior of the composites are also important. It was considered by Lou et al. [16] based on the fiber diamond distribution model in the original multiscale framework. However, the fiber random distribution model and interface debonding have not been considered simultaneously in the multiscale framework.

For the homogenization process, different methods were adopted. Li et al. [14], Liu et al. [15] and Lou et al. [16] adopted the sudden degradation model, which means that, once a certain damage mode is activated, the macroscale properties are degraded with a certain percentage suddenly. Lou et al. [16] believed that, once the interface debonding occurs, the matrix damage or fiber breakage is detected and a certain damage value is set for the macroscale element. To consider more microscale information, in Xu et al. [13], instead of degrading the macroscale elastic parameters directly, the elastic parameters of the constituents are degraded based on the damage states. Then, the analytical model for elastic parameter prediction of unidirectional laminates is used for the prediction of the elastic parameters of macroscale models. However, in this homogenization method, an assumption is made that the equivalent damage value of a matrix can be obtained through the volume average method. To exclude the assumption, the neural-network-based degradation method will be developed in this study to obtain the equivalent elastic parameters precisely.

In this study, the MMF-based multiscale method is modified with the clustering method so that the fiber random distribution model considering the interface can be considered at the microscale. Besides, based on the microscale RVE discretized with clusters, a new degradation rule is developed with the feed-forward neural network. In the first section of this study, the finite element models for the open-hole laminates to be analyzed are introduced. In the second section, details for the modified multiscale method are shown systematically. In the last section, the predicted results are illustrated and compared with the experimental results. The effects of interface debonding, degradation rules and fiber distribution patterns are also revealed.

## 2. Multiscale Finite Element Modeling

### 2.1. Macro Model

**Figure 1:**
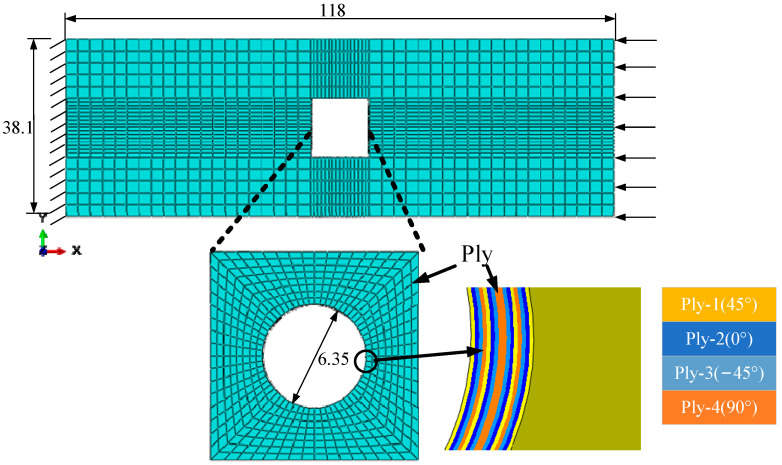
Finite element discretization for the macroscale model.

**Figure 2:**
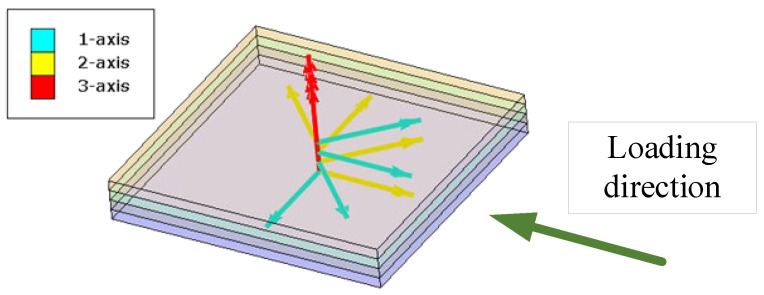
Local orientation for the first four plies.

The model analyzed in Refs. [14,16] is adopted in this study to validate the efficiency and accuracy of the multiscale method to be modified. The open-hole laminate [45/0/−45/90]_2s_ shown in Figure 1 was analyzed under compressive loading. In Figure 1, four different colors refer to different plies. The orientation of first four plies with respect to the loading direction is shown in Figure 2. The 1-axis refers to the fiber direction. According to the orientation between the fiber direction and loading direction, the first ply is set to be 45° ply, the second ply is set to be 0°, the third ply is set to be −45° and the fourth ply is set to be 90°. The size of the open-hole laminates is 118 × 38.1 × 1.6 mm^3^ and the diameter of the hole is 6.35 mm. More details about the macroscale model can be referred to in Ref. [14]. The model is discretized with C3D8R elements in ABAQUS and the mesh around the hole is refined to describe the stress concentration (Figure 1). One element is used through the ply thickness to represent each layer, and this assumption is widely used [17].

### 2.2. Micro Model Considering Interfaces

**Figure 3:**
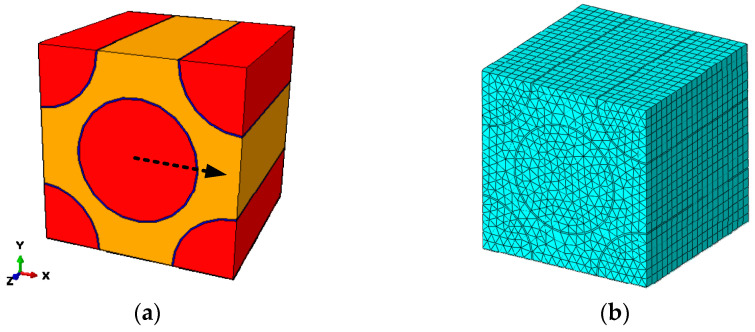
Fiber diamond distribution microscale RVE considering interface. (**a**) geometrical model; (**b**) finite element model.

In Ref. [14], the fiber arrangement is assumed to be regular and only the fiber and matrix are considered at the microscale. The diameter of the carbon fiber is around 7 μm. The fiber volume fraction is 56%. To evaluate the effects of the interface, the microscale RVE considering interface is established according to Ref. [16] in Figure 3. The ratio between the thickness of interface and the radius of the fiber is 1/35, so the thickness of the interface is set to 0.5 μm. There are some studies revealing that the interfaces are heterogeneous and the modulus of the interface close to the fiber is larger than that close to the matrix [19,20]. In this study, the interface is assumed to be homogeneous, and the assumption was adopted in Ref. [16] to reveal the effects of interface debonding on the failure analysis of the open-hole laminates under compressive loading. The equivalent elastic modulus of the interface is also referred to from Ref. [16]. The finite element model is also shown in Figure 3. For the fiber and matrix, the C3D6 elements in ABAQUS are adopted to discretize the RVE, and, for the interface, the C3D8R elements are adopted.

Actually, the fiber arrangement pattern at the microscale is random, and it has been proved that the elastic and inelastic parameters predicted from the fiber random distribution models are closer to the experimental results [21,22]. Thus, the fiber random distribution model is considered in this study to reveal the coupling effects of interface and fiber arrangement patterns on the compressive strength of the open-hole laminates. The generation method for the fiber random distribution models in Ref. [23] is adopted in this study, which is modified from the random perturbation method. Through combining the fiber perturbation and global crisscrossing method, the fiber random distribution model can be established efficiently.

**Figure 4:**
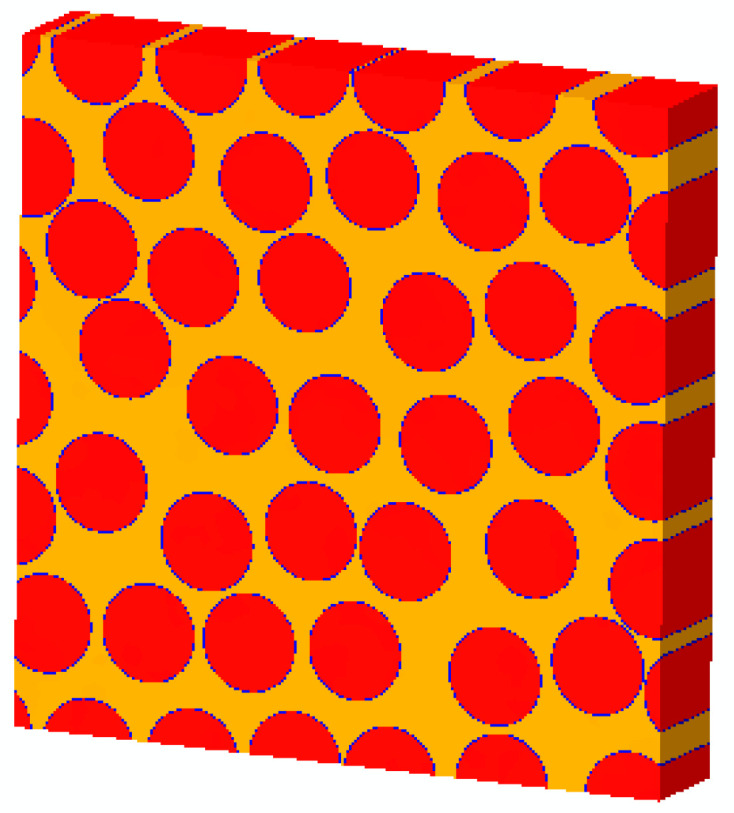
Fiber random distribution microscale RVE considering interface.

In the first step, according to the desired fiber volume fraction (56%), the model with fiber square distribution is established [24]. Then, in one iteration, global crisscrossing of the position of each fiber is conducted to improve the efficiency of the generation of fiber random distribution model [25]. After the global crisscrossing, the limit of movement in the x and y direction of each fiber is determined and the random perturbation of each fiber position is conducted. Then, a new position for each fiber can be obtained randomly under the conditions that there is non-overlapping between each two fibers. After 100 iterations adopted in this study, the model with fiber random distribution can be obtained. The fiber random distribution RVE model considering interface is shown in Figure 4.

## 3. Multiscale Model Considering Temperature Effects

### 3.1. Calculation of Amplification Factors

To calculate the stress values in the microscale RVE based on the stress/strain values in one macroscale element, the amplification factors are adopted [12,13] as shown in the Equation 1. To obtain the amplification factors, the periodical boundary conditions should be applied to the microscale RVE first. Then, six different loading cases are considered for the microscale elastic analyses. For one loading condition, the strain value *ε_i_* (*i* = 1, 2, 3 … or 6) is set to be one and the other strain values are set to be zero. Thereafter, the six equivalent stress values derived from the RVE under one loading case are the *i*-th column elements in the amplification matrix. At last, the amplification factors for the one microscale element can be obtained. There are different kinds of amplification factors [18], and the strain–stress amplification factors [17] (Equation (1)) are adopted to transfer the macroscale information to the microscale model in this study.

(1) $$\left\{\begin{array}{c} \sigma_{1}\\ \sigma_{2}\\ \sigma_{3}\\ \sigma_{4}\\ \sigma_{5}\\ \sigma_{6} \end{array}\right\}=\left[\begin{array}{cccccc} A_{11} & A_{12} & A_{13} & A_{14} & A_{15} & A_{16}\\ A_{21} & A_{22} & A_{23} & A_{24} & A_{25} & A_{26}\\ A_{31} & A_{32} & A_{33} & A_{34} & A_{35} & A_{36}\\ A_{41} & A_{42} & A_{43} & A_{44} & A_{45} & A_{46}\\ A_{51} & A_{52} & A_{53} & A_{54} & A_{55} & A_{56}\\ A_{61} & A_{62} & A_{63} & A_{64} & A_{65} & A_{66} \end{array}\right]\left\{\begin{array}{c} {\bar{\varepsilon }}_{1}\\ {\bar{\varepsilon }}_{2}\\ {\bar{\varepsilon }}_{3}\\ {\bar{\varepsilon }}_{4}\\ {\bar{\varepsilon }}_{5}\\ {\bar{\varepsilon }}_{6} \end{array}\right\}$$

As the microscale elastic analyses are conducted at the global coordinates, the calculated stress values can be used to obtain amplification factors of the fiber and matrix directly. However, for the interface, instead of using the stress components in the global coordinates, the tractions in the local coordinates are usually used to judge the interface debonding, including the normal traction (*t_n_*), longitudinal shear traction (*t_x_*) and tangential shear traction (*t_s_*) [19]. As the traction forces are used for the judgment of interface debonding, the strain–stress amplification factors for the interface should be replaced with the strain–traction amplification factors. It is realized with Equation (2) [26]. The process for deriving the equation can be found in Appendix A.

(2) $$\left[\begin{array}{c} t_{n}\\ t_{x}\\ t_{s} \end{array}\right]=\left[\begin{array}{cccccc} 0 & A_{12} & A_{13} & 0 & 0 & A_{16}\\ 0 & 0 & 0 & A_{24} & A_{25} & 0\\ 0 & A_{32} & A_{33} & 0 & 0 & A_{36} \end{array}\right]\left[\begin{array}{c} {\bar{\varepsilon }}_{1}\\ {\bar{\varepsilon }}_{2}\\ {\bar{\varepsilon }}_{3}\\ {\bar{\varepsilon }}_{4}\\ {\bar{\varepsilon }}_{5}\\ {\bar{\varepsilon }}_{6} \end{array}\right]$$

### 3.2. Extraction of Strain–Stress Amplification Factors with Clustering Analysis

**Figure 5:**
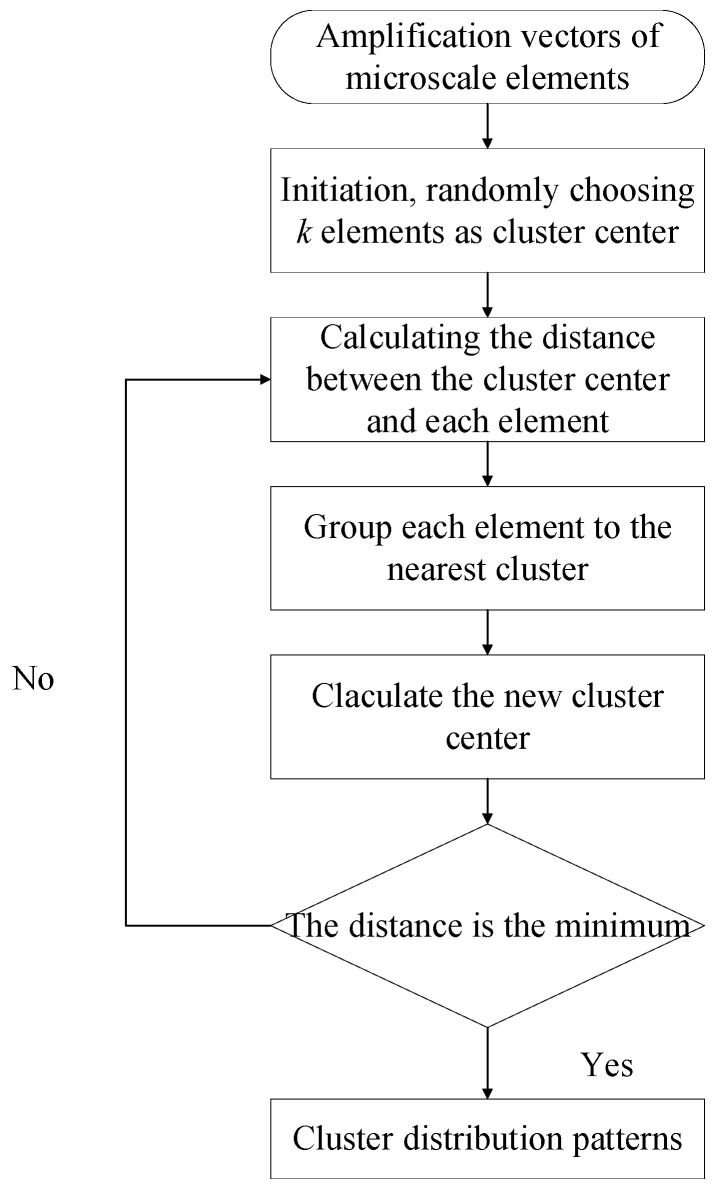
Flowchart for deriving cluster distribution patterns.

To save the computational capability and reserve the geometrical characteristics of the microscale RVE during the multiscale analysis, the clustering method is adopted to discretize the RVE, and the elements in one cluster are believed to have almost the same mechanical behaviors under external loadings [27,28,29]. For the fiber and matrix, the clustering analysis is conducted based on the amplification factors obtained from the elastic analysis. The clustering analysis procedure is the same as that adopted in Ref. [18]. The flowchart for deriving the clustering distribution patterns of the microscale RVE is shown in Figure 5. First, the matrix in Equation (3) [18] is assembled with the amplification factors.

(3) $$\left[\begin{array}{c} \vdots \\ A_{11}^{i},\cdots A_{16}^{i},A_{21}^{i},\cdots A_{26}^{i},\cdots A_{66}^{i}\\ \vdots \end{array}\right]$$

A row of the matrix is expressed as a vector $B^{i}=\left[A_{11}^{i},\ldots A_{16}^{i},A_{21}^{i},\ldots ,A_{26}^{i},\ldots A_{66}^{i}\right]$, which presents the stress/strain values in the element *i* under the six mechanical loading conditions. Then, according to the cluster number, *k* elements are chosen randomly for each cluster as its center. Thereafter, each B finds out the center that it is closest to with Equation (4) (Ref. [27]). Next, the new centroid of the elements related with the previous center should be found. This process needs to be repeated until the distance (*Dis*) is the minimum (Equation (5) from Ref. [27]), which groups each element into one cluster.

(4) $${\left\|\left\{B^{i}\right\}-{\bar{B}}^{N}\right\|}^{2}<{\left\|\left\{B^{i}\right\}-{\bar{B}}^{M}\right\|}^{2}\;\;\;\forall M,M\neq N$$

where *M* and *N* represent the cluster number.

(5) $$Dis=\arg\min{\sum_{J=1}^{k}\sum_{i\in S^{J}}\left\|B^{i}-{\bar{B}}^{J}\right\|}^{2}$$

where *S^J^* represents the *J*-th cluster, *i* is the element number and *J* is the cluster number.

More details about the clustering process can be referred to in Refs. [27,28,29]. The amplification factors of the clusters are believed to be representative of the amplification factors of all elements in the RVE [18] and would be used to transfer the macroscale information to the microscale. For the interface, the clustering analysis is conducted based on the strain–traction amplification factors, so the interface elements in one cluster would have almost the same traction values under external loading conditions.

### 3.3. Failure Criteria of the Constituents

After the stress values in the constituents at the microscale are obtained, the damage initiation can be judged based on the failure criteria. For the fibers, the maximum stress criteria are adopted, which indicates that, once the stress along the longitudinal direction exceeds the strength value, the fiber elements are failed. As the tensile (*T_f_*) and compressive (*C_f_*) strength values of the fibers are different, the failure criteria for the fibers are composed of two parts (Equation (6)) [16].

(6) $$\begin{array}{l} \frac{\sigma_{11}^{T}}{T_{f}}\geq 1\\ -\frac{\sigma_{11}^{C}}{C_{f}}\geq 1 \end{array}$$

Due to the differences in the tensile and compressive strength of the matrix, the failure criteria for the matrix are also composed of two parts (Equation (7)) [16]. If the matrix is under the tensile loading condition, the damage initiation is judged by comparing the first stress invariant with the tensile strength of the matrix. If the matrix is under the compressive loading condition, the damage is activated by comparing the equivalent stress with the compressive strength.

(7) $$\begin{array}{l} \frac{I_{1}^{m}}{T_{m}}\geq 1\\ \frac{\sigma_{vm}^{m}}{C_{m}}\geq 1 \end{array}$$

where $I_{1}^{m}=\sigma_{11}+\sigma_{22}+\sigma_{33}$ and $\sigma_{vm}^{m}=\sqrt{0.5\left[{\left(\sigma_{11}-\sigma_{22}\right)}^{2}+{\left(\sigma_{11}-\sigma_{33}\right)}^{2}+{\left(\sigma_{22}-\sigma_{33}\right)}^{2}+6\left(\tau_{12}^{2}+\tau_{23}^{2}+\tau_{13}^{2}\right)\right]}$.

To judge the initiation of interface debonding, the failure criteria shown in Equation (8) [16] are adopted.

(8) $$\left\{{\left(\frac{\langle t_{n}\rangle }{N}\right)}^{2}+{\left(\frac{t_{x}}{S}\right)}^{2}+{\left(\frac{t_{s}}{S}\right)}^{2}\right\}\geq 1$$

where *t_n_* and *t_x(s)_* are interfacial normal traction and interfacial shear traction, respectively, which are highly important for the determination of whether interface debonding will initiate. < > is Macaulay bracket, *N* is the normal strength and *S* is the shear strength of the interface.

In this study, the elastic and strength values for the fiber, matrix and interface are referred to in Refs. [14,16] and shown in Table 1 and Table 2.

### 3.4. Damage Model Derivation

Once interface debonding, matrix damage or fiber breakage is activated, certain damage values are calculated and the flexibility matrix is degraded according to Refs. [16,26] (Equation (9)). It can be found that the damage of the matrix and interface contributes to the degradation of the transverse elastic modulus. The damage of the fiber results in the degradation of the longitudinal elastic modulus. The degraded stiffness values of the macroscale element induced by its institutes’ failure are shown in Equation (10) (Ref. [16]). In Ref. [16], the sudden degradation model is adopted. It means that, if the interface debonding at any critical point is activated and the matrix is intact, the damage parameter *d_m,i_* equals 0.11 and, if both interface debonding and matrix damage initiate, the *d_m,i_* is set to 0.99. If the fibers are invalid, the damage parameter *d_f_* is set to 0.99.

(9) $${\left[S\right]}_{ijkl}=\left[\begin{array}{cccccc} \frac{1}{E_{1}\left(1-d_{f}\right)} & -\frac{v_{12}}{E_{1}} & -\frac{v_{13}}{E_{1}} & & & \\ & \frac{1}{E_{2}\left(1-d_{m,i}\right)} & -\frac{v_{23}}{E_{2}} & & & \\ & & \frac{1}{E_{3}\left(1-d_{m,i}\right)} & & & \\ & & & \frac{1}{G_{12}\left(1-d_{f}\right)\left(1-d_{m,i}\right)} & & \\ & & & & \frac{1}{G_{13}\left(1-d_{f}\right)\left(1-d_{m,i}\right)} & \\ & & & & & \frac{1}{G_{23}\left(1-d_{f}\right)\left(1-d_{m,i}\right)} \end{array}\right]$$

(10) $$\begin{array}{l} C_{11}=E_{1}\left(1-d_{f}\right)\left[1-{\left(1-d_{m,i}\right)}^{2}v_{23}^{2}\right]/A\\ C_{22}=E_{2}\left(1-d_{m,i}\right)\left[1-\left(1-d_{m,i}\right)\left(1-d_{f}\right)v_{13}v_{31}\right]/A\\ C_{33}=E_{2}\left(1-d_{m,i}\right)\left[1-\left(1-d_{m,i}\right)\left(1-d_{f}\right)v_{12}v_{21}\right]/A\\ C_{12}=E_{2}\left(1-d_{m,i}\right)\left(1-d_{f}\right)\left[\left(1-d_{m,i}\right)v_{13}v_{23}+v_{12}\right]/A\\ C_{13}=E_{2}\left(1-d_{m,i}\right)\left(1-d_{f}\right)\left[\left(1-d_{m,i}\right)v_{12}v_{23}+v_{13}\right]/A\\ C_{23}=E_{2}{\left(1-d_{m,i}\right)}^{2}\left[\left(1-d_{f}\right)v_{12}v_{31}+v_{23}\right]/A\\ C_{44}=G_{12}\left(1-d_{m,i}\right)\left(1-d_{f}\right)\\ C_{55}=G_{13}\left(1-d_{m,i}\right)\left(1-d_{f}\right)\\ C_{66}=G_{23}\left(1-d_{m,i}\right)\left(1-d_{f}\right)\\ A=1-\left(1-d_{m,i}\right)\left(1-d_{f}\right)v_{12}v_{21}-{\left(1-d_{m,i}\right)}^{2}v_{23}v_{32}-\\ \;\left(1-d_{m,i}\right)\left(1-d_{f}\right)v_{13}v_{31}-2{\left(1-d_{m,i}\right)}^{2}\left(1-d_{f}\right)v_{12}v_{31}v_{23} \end{array}$$

For the fiber breakage, it had been demonstrated that, once the fiber breakage is initiated, the bearing capability of the macroscale element along the longitudinal direction is lost suddenly [13,17]. Thus, in this study, the sudden degradation rule is adopted along the longitudinal direction. However, instead of using the sudden degradation model to describe the failure of interface and matrix, a feed-forward-neural-network-based degradation rule is developed in this study. With this method, the effects of arbitrary failure patterns of matrix and interface on the *d_m,i_* can be evaluated quantitatively rather than empirically with a critical value.

The feed-forward neural network (FFNN) is composed of a layer of input neurons, a layer of output neurons and one or more layers of hidden neurons [30]. Each hidden layer has different numbers of neural elements. Information flows from one layer to the other layers in a feed-forward manner. Neurons in each layer are fully interconnected to preceding and subsequent layer neurons, with each interconnection having an associated connection weight. The network function is determined largely by the connections between the elements. The nonlinear activation function is used in the hidden and output layers’ neurons, which is used to ensure that the computer simulations are non-linear. To have a neuron network perform a particular function, the values of the connections (weights) between the elements should be trained and adjusted correctly.

**Figure 6:**
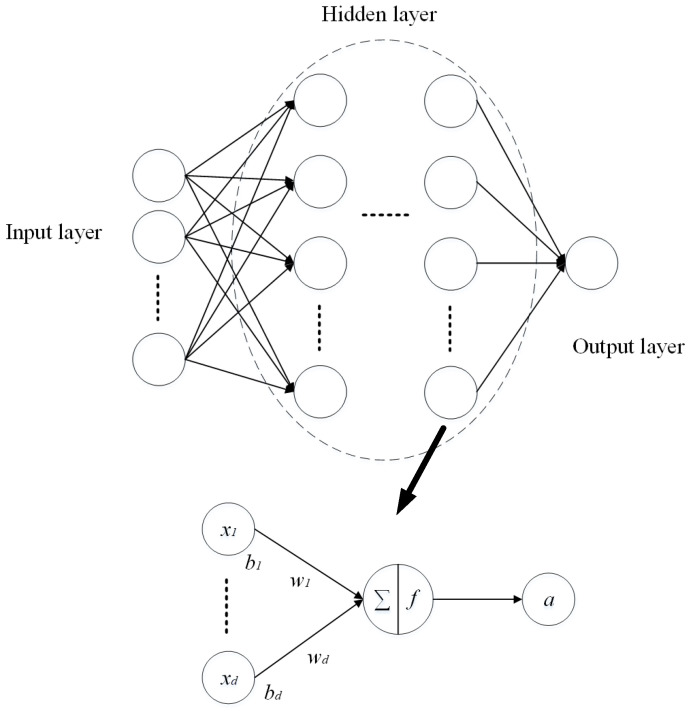
Schematic description of the FFNN.

The schematic description of the FFNN is shown in Figure 6. Hidden layers can contain one or several layers for the practical application. The relationship between the input vector *X* = [*x*_1_, *x*_2_,…, *x_d_*]^T^ and output vector *a* of one neural element can be described in Equation (11) [30] and schematically drawn in Figure 5.

(11) $$a=f\left(W^{T}X+B\right)$$

where *W* = [*w*_1_, *w*_2_,…, *w_d_*] is the weights of the preceding layer neurons and *B* = [*b*_1_, *b*_2_,…,*b_d_*] is the bias vector for inputs. The *f* is the non-linear active function. Commonly used activation functions are Tanh, ReLU, Sigmoid, Softplus and Linear, etc. [30].

After the network is established, it has to be trained to adapt to the given problem using a large dataset that was generated a priori. The training or learning process involves adjusting the weights and bias of the network to approximate closely the outputs of the training dataset. The error back propagation algorithm is adopted to obtain the optimal weight and bias values for the neurons, with a least sum squared optimality criterion of errors between the predicted and desired values. More details about the FFNN and its training algorithm can be referred to in Refs. [31,32].

In this study, to establish the connection between the interface and matrix clusters’ failure patterns and the equivalent transverse elastic modulus, the first step is to establish the dataset. To obtain the equivalent elastic modulus of the RVE with arbitrary matrix and interface clusters failed, the elastic prediction method presented in Ref. [18] is adopted. If one cluster is set to be failed, the elastic modulus is assumed to be a very small value. Six independent loading cases are applied on the RVE and the equivalent elastic parameters can be obtained. If the matrix and interface regions are discretized with *n* clusters and *m* clusters, respectively, there are 2^(*n*+*m*)^ kinds of failure patterns. The input vector for the network has *n* + *m* elements and each one has two equally possible values, 0 or 1, which indicate the integrity and failure of the cluster, respectively. The output of the FFNN is the equivalent elastic modulus value. Through comparing the equivalent elastic parameters with the intact elastic parameters, the equivalent damage value induced by the failure of matrix and interface can be investigated [33] (Equation (12)).

(12) $$d_{m,i}=1-\frac{E_{damage}}{E_{intact}}$$

In Refs. [34,35], it has been demonstrated that every bounded continuous function can be approximated with arbitrarily small error by network with one hidden layer. The Sigmoid activation function is adopted in this study. Thus, to establish the FFNN model for elastic modulus prediction, the only problem is to determine the number of neurons in the hidden layer. There are some empirical functions that were developed to obtain the number based on trial-and-error calculations, and they are shown in Equation (13) [36]. To evaluate the performance of the trained FFNN, the evaluation equations are shown as the root mean square error (RMSE) and the coefficient of determination (*R*^2^) [36]. The RMSE is used to evaluate the errors (Equation (14) from Ref. [36]), which means that, if the RMSE is closer to zero, the model error is smaller. The *R*^2^ is also used to evaluate the accuracy of the model (Equation (15) from Ref. [36]), and, if the *R*^2^ is closer to one, the model is more accurate.

(13) $$\begin{array}{l} H=\sqrt{I+O}+N\\ H=\sqrt{I\times O}\\ H=\log_{2}O \end{array}$$

where *I* is the number of the input vector elements, *O* is the number of the output vector elements and *N* is the number between 1 and 10.

(14) $$RMSE=\sqrt{\frac{1}{m}\sum_{i=1}^{m}{\left(y_{i}-{\hat{y}}_{i}\right)}^{2}}$$

(15) $$R^{2}=1-\frac{\sum_{i=1}^{m}{\left(y_{i}-{\hat{y}}_{i}\right)}^{2}}{\sum_{i=1}^{m}{\left(y_{i}-{\bar{y}}_{i}\right)}^{2}}$$

where $y_{i}$ is the actual value, ${\hat{y}}_{i}$ is the value predicted based on FFNN, ${\bar{y}}_{i}$ is the mean value of the actual values and *m* is the number of cases for the train.

### 3.5. Modified Multiscale Numerical Implementation

**Figure 7:**
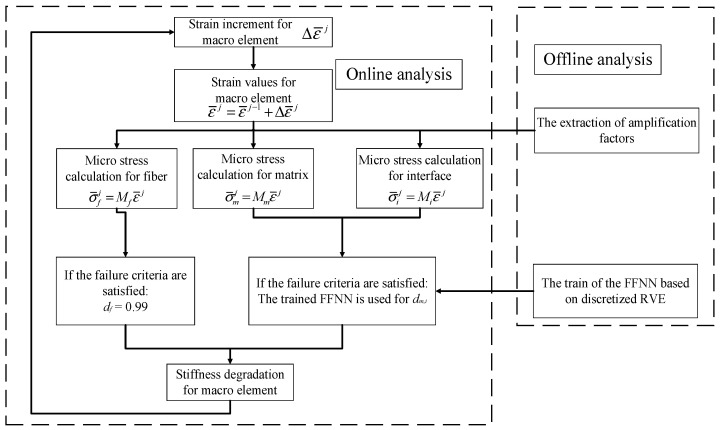
Analysis flow of the modified MMF-based multiscale method.

The open-hole laminates are analyzed based on the modified multiscale method to reveal the effects of the complex internal structures and the developed degradation rules on the predicted compressive behaviors (Figure 7). First, the elastic analysis and the clustering analysis are conducted for the microscale RVEs so that the amplification factors for the fiber, matrix and interface are obtained. Then, with the discretized RVE, the relation between the failure patterns of the RVE and the equivalent transverse elastic modulus is established based on the FFNN. At last, the online analysis for the open-hole laminates is conducted.

For the online analysis, the strain values in the macroscale element are calculated first. Then, they are used to obtain the stress values for the fiber and matrix clusters and obtain the traction values for the interface clusters. Thereafter, a damage initiation judgment should be conducted. If the failure criteria of the fibers are satisfied, the damage value *d_f_* for the fiber is set to be 0.99. For the matrix and interface, if the failure criteria for each constituent are reached, the equivalent transverse modulus is derived and compared with the intact value to obtain the damage value *d_m,i_* in the transverse direction. At last, with the two damage values, the stiffness matrix of the macroscale element is degraded and used for the next analysis step.

## 4. Results and Discussion

### 4.1. Offline Analysis

**Figure 8:**
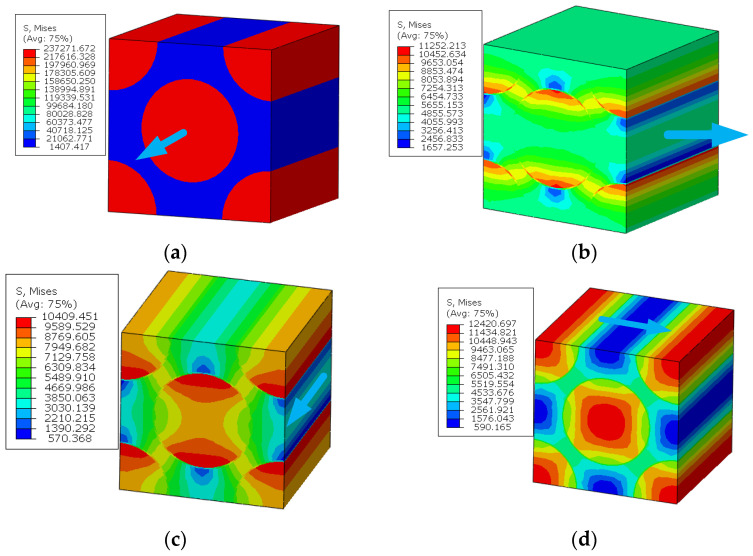
Stress distribution patterns for the fiber diamond distribution model. (**a**) longitudinal tensile loading; (**b**) transverse tensile loading; (**c**) longitudinal shear loading; (**d**) transverse shear loading.

**Figure 9:**
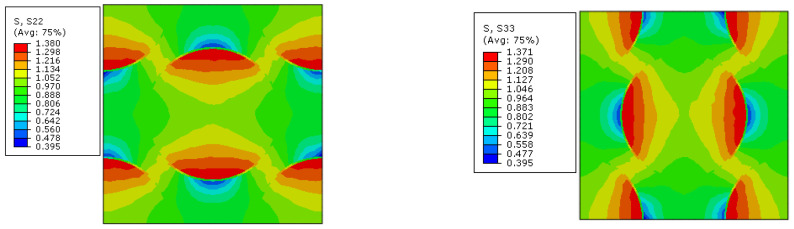
Stress distribution patterns for the model under unit stress loading along transverse direction.

**Figure 10:**
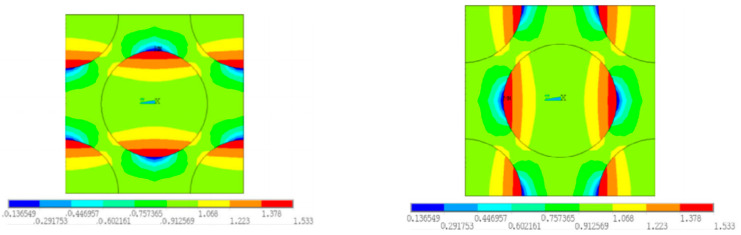
Stress distribution patterns for the model under unit stress loading along transverse direction in Ref. [37].

As the analysis results from Refs. [14,16] are used to validate the modified multiscale model, the geometrical parameters of the model from Refs. [14,16] are adopted here for comparison. Thus, the RVE with a fiber diamond distribution pattern is analyzed under six independent strain loading conditions. The longitudinal tensile, transverse tensile, longitudinal shear and transverse shear stress values are shown in Figure 8. It can be found that the stress concentrates around the fibers and, due to the symmetry of the RVE, the stress distribution patterns are also symmetric. With the same FEM model and boundary conditions, the stress distribution patterns under the unit stress loading are shown in Figure 9. The stress distribution patterns are similar with those (Figure 10) in Ref. [37]. The consistency between them verifies the accuracy of the elastic analysis method and results in this study. It should be noted that the maximum stress value predicted in this study is smaller than that from Ref. [37], which should be attributed to the consideration of interface in this study.

**Figure 11:**
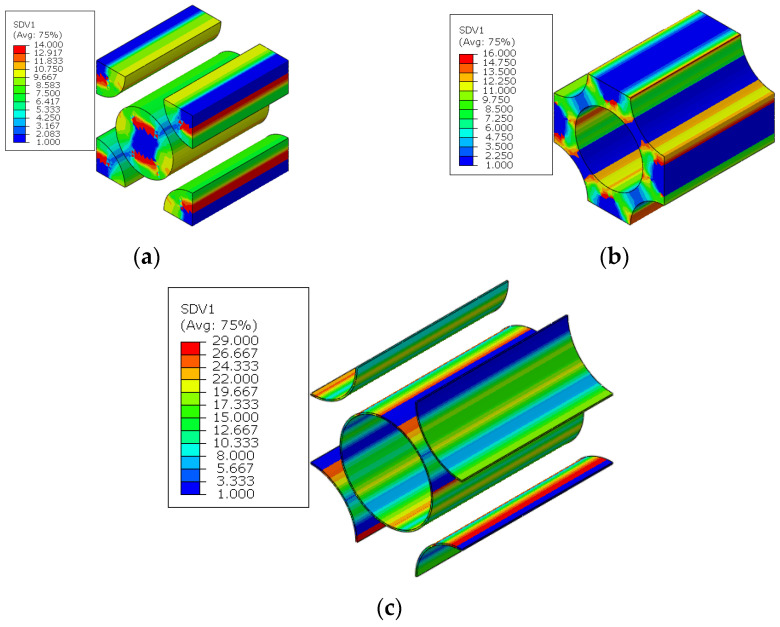
Cluster distribution pattern for the fiber diamond distribution model. (**a**) fiber cluster; (**b**) matrix cluster; (**c**) interface cluster.

For the fiber diamond distribution patterns, the cluster distribution patterns for the fiber, matrix and interface are shown in Figure 11. The chosen cluster numbers of the fiber and matrix are 14 and 16, respectively, which have been demonstrated in Ref. [18]. For the cluster number of interfaces, the chosen value is 30, which is larger than the number of reference points in the Ref. [16]. It can be found that the cluster distribution patterns for the matrix and fiber regions are almost symmetrical, which should be attributed to the symmetry of the RVE. For the interface region, the interface elements in one cluster have almost the same orientation angle.

**Figure 12:**
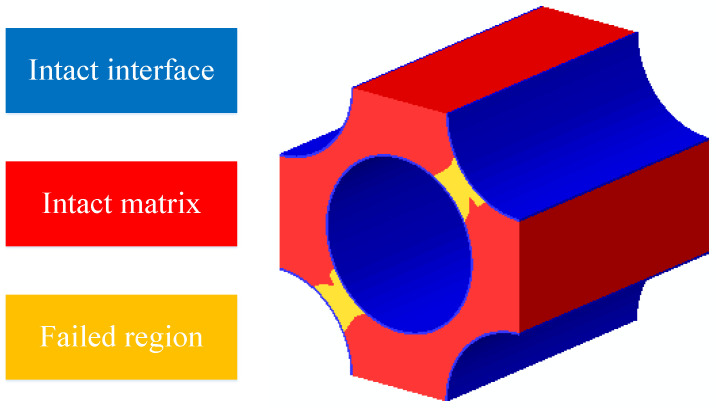
Illustration for one failure pattern of matrix and interface.

**Figure 13:**
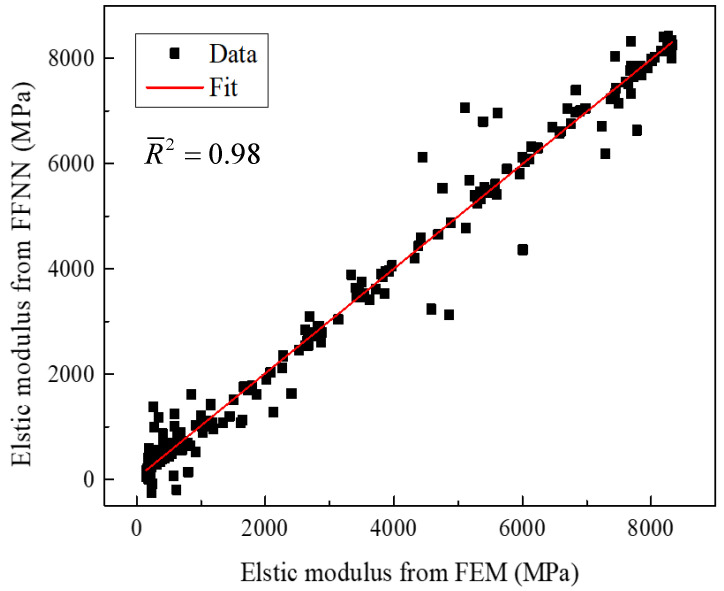
Linear regression of the elastic modulus between those from the FFNN and FEM.

Based on the RVE discretized with clusters, certain interface and matrix clusters are randomly chosen and are set to be failed. An illustration for one failure pattern is shown in Figure 12. Then, the equivalent transverse elastic modulus is derived through the elastic analyses of the degraded RVEs. Further, 200 cases are analyzed and used to establish the connection between the failure patterns and the equivalent elastic modulus. Among them, 140 cases are used to train the network (training), 30 cases are used to validate the network (validation) and 30 cases are used to test the trained network (testing). Through the trial-and-error calculations based on the FFNN, the number of neurons in the hidden layer is set to be 7, and the performance of the trained network is shown in Table 3. It can be found that the RMSE values are smaller compared with the intact transverse elastic modulus (10 GPa). For the *R*^2^, they are all very close to one. The regression line comparing the values from the FEM and the values from the FFNN is shown in Figure 13, and it can be found that the slope of the regression line is very close to one. Based on the conclusions derived above, the accuracy of the chosen neuron number and the effectiveness of the FFNN are demonstrated.

**Figure 14:**
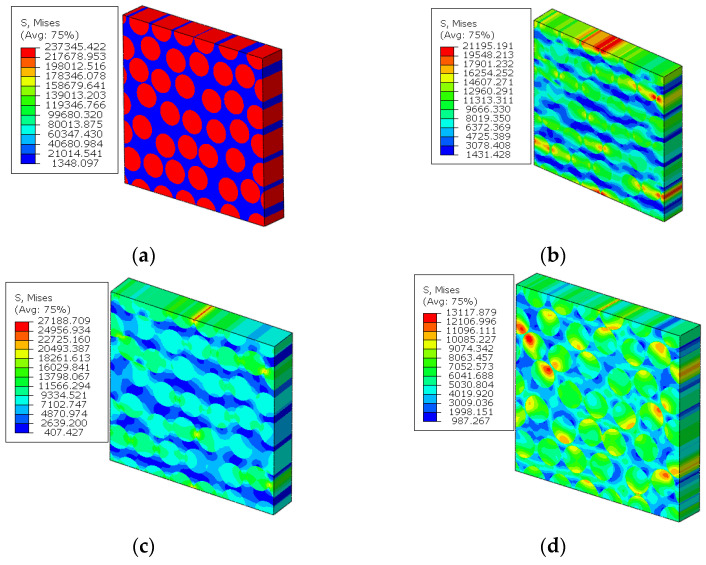
Stress distribution patterns for the fiber random distribution model. (**a**) longitudinal tensile loading; (**b**) transverse tensile loading; (**c**) longitudinal shear loading; (**d**) transverse shear loading.

**Figure 15:**
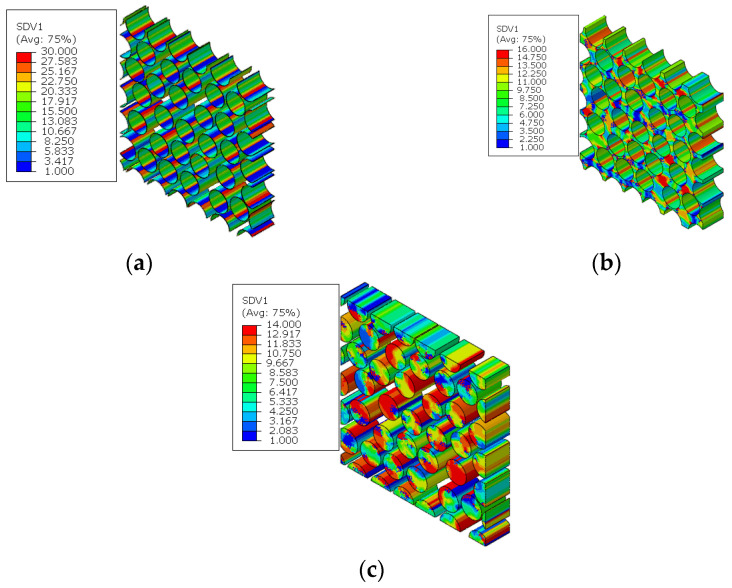
Cluster distribution pattern for the fiber diamond distribution model. (**a**) interface cluster; (**b**) matrix cluster; (**c**) fiber cluster.

The stress distribution patterns for the fiber random distribution model are shown in Figure 14. It can be found that smaller inter-fiber distance contributes to much larger stress concentration in the fiber random distribution model compared with the fiber diamond distribution model. The clustering analysis for the fiber random distribution models is also conducted, and the obtained cluster distribution patterns are shown in Figure 15. It can be found that the stress concentration regions are almost in one cluster. Then, based on the discretized fiber random model, the relation between one certain failure pattern and the transverse elastic modulus is established and 200 cases are analyzed. The FFNN is trained, validated and tested with the 200 cases. The number for the training, validation and testing is the same with that used for the fiber diamond distribution model. The performance of the trained FFNN is shown in Table 4, which demonstrates the effectiveness of the trained network.

### 4.2. Online Analysis

#### 4.2.1. Model Validation

**Figure 16:**
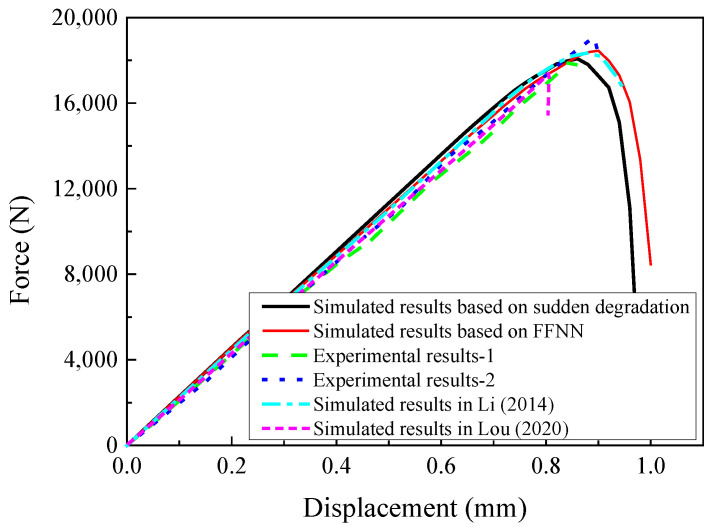
Predicted compressive responses of the open-hole laminates.

To validate the multiscale analysis procedure, the macroscale models are analyzed considering the fiber diamond distribution pattern, and the obtained results will be compared with the experimental and simulated results from Ref. [16]. With the amplification factors for the constituents, the compressive behaviors of the open-hole laminates are predicted and compared with the experimental results in Refs. [14,16] (Figure 16). All strength values in Figure 16 are listed in Table 5. It can be found that the strength values predicted in this study are close with the experimental results, which demonstrates the effectiveness of the multiscale analysis method. Besides, the effects of the sudden degradation rule or FFNN-based degradation rule are also compared in Figure 16. It can be found that the reaction force value at displacement 0.6 mm based on the FFNN degradation method (13.2 KN) is smaller than that obtained from the model based on the sudden degradation method (13.6 KN). However, the compressive strength predicted from the model based on the FFNN method (18.5 KN) is larger than that predicted from the model based on the sudden degradation method (18.0 KN), and the percentage increase is 2.8%.

**Figure 17:**
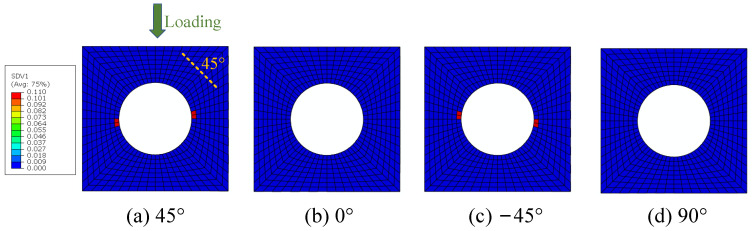
The initiations regions for fiber–matrix interface debonding.

The constituents’ failure modes, including fiber–matrix interface debonding, matrix damage and fiber breakage, are identified from the damage initiation to final failure with the modified MMF-based multiscale method. The interface debonding initiation regions based on the sudden degradation model are located at the hole edge in the transverse direction of the 45° and −45° plies (Figure 17), which is the same with that obtained from Ref. [16]. The critical displacement loading value for the interface debonding is 0.06 mm. Compared with the model without considering the interface [14], it can be found that the damage initiates much earlier due to the interface debonding.

**Figure 18:**
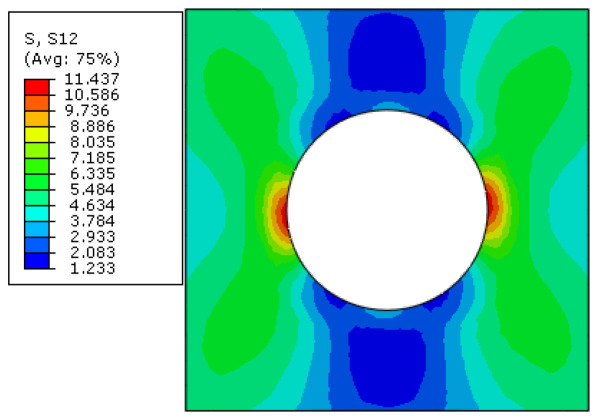
Intralaminar shear stress distribution pattern.

Before the initiation of interfacial debonding, the intralaminar shear stress (*τ*_12_) distribution pattern in the 45° ply is shown in Figure 18. The stress distribution pattern is similar with that obtained in Ref. [16], in which it is regarded as the primary reason for the interfacial debonding. However, it is difficult to determine the damage initiation and propagation processes at the microscale with the stress distribution patterns at the macroscale.

In the analyses conducted in Refs. [3,4,5,6,7,8,9,10,11], only the damage propagation processes at the macroscale were obtained and analyzed. With the modified MMF multiscale method developed in this study, besides the obtained macroscale results, the microscale information can also be obtained. With the amplification factors, the failure indices and stress states of the constituents are obtained and mapped into the microscale RVE, which would be helpful for revealing the failure mechanisms more clearly.

**Figure 19:**
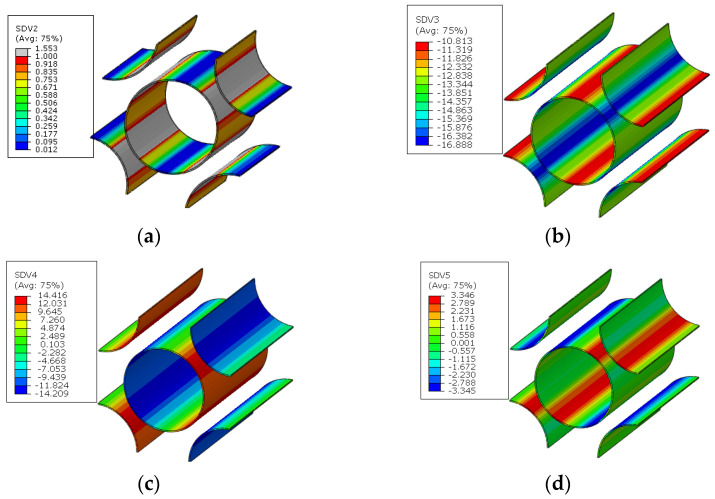
Failure indices and traction force states for the interface. (**a**) failure indices; (**b**) normal traction; (**c**) longitudinal shear traction; (**d**) tangential shear traction.

**Figure 20:**
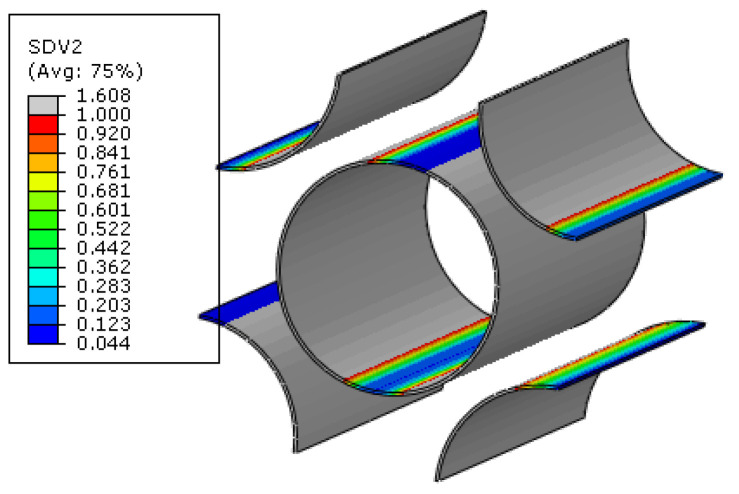
Damage propagation in the interface of fiber diamond distribution model.

For the macroscale element where the interface debonding initiates first in the 45° ply, the failure indices and the traction distribution patterns in the corresponding interface regions are shown in Figure 19. It can be found that, in the normal direction, the compressive traction is subjected. Based on the interface debonding criteria, it can be concluded that the interfacial debonding at the location shown in Figure 17 is independent on the normal traction value. Comparing the longitudinal shear traction with tangential shear traction distribution patterns, it can be found that it is the longitudinal shear traction that makes the most contribution to the interface failure. From the distribution pattern of failure indices, it can be found that the interface debonding initiates uniformly due to the interaction of the fibers. Besides the damage initiation position, the damage propagation process at the microscale can also be obtained. With the increase in displacement loading, the interface debonding regions increase quickly at the microscale (Figure 20).

**Figure 21:**
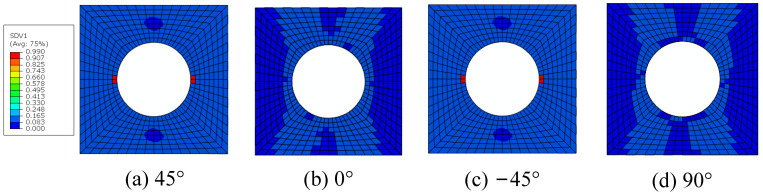
The initiations regions for matrix damage.

**Figure 22:**
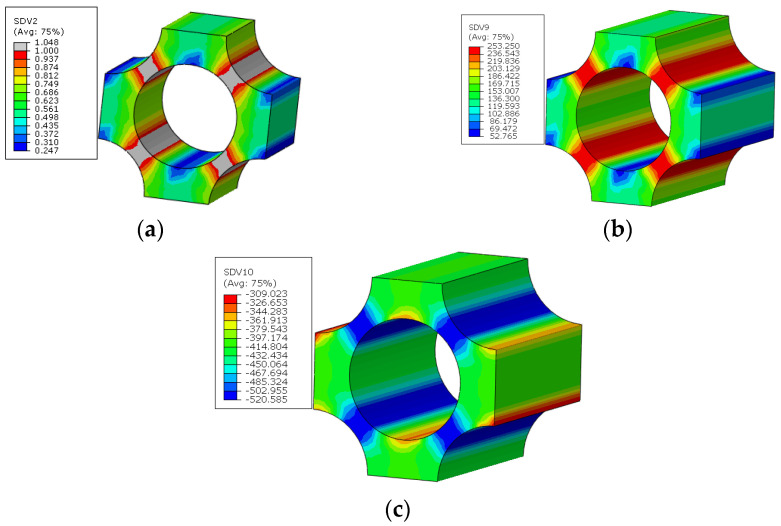
Failure indices and stress states in the matrix for fiber diamond distribution model. (**a**) failure indices; (**b**) equivalent stress; (**c**) first stress invariant.

**Figure 23:**
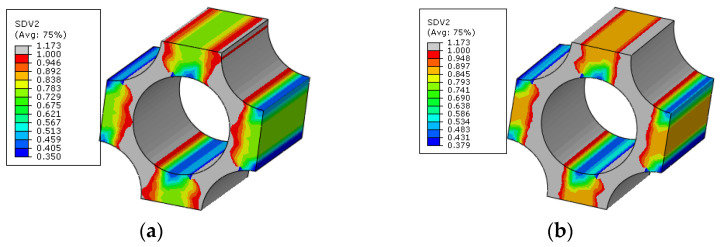
Matrix damage propagation in the matrix for fiber diamond distribution model. (**a**) at displacement loading 0.66mm; (**b**) at displacement loading 0.7 mm.

With the increase in the external loading, the matrix damage also initiates at the hole edge in transverse direction of the 45° and −45° plies (Figure 21), and the critical displacement loading for the matrix damage initiation is 0.64 mm. With the modified MMF multiscale method, for the macroscale element where the matrix damage initiates first in the 45° ply, the failure indices and stress distribution patterns at the microscale are shown in Figure 22. It can be found that the matrix is under compressive states, so it is proved that matrix damage is induced by the von Mises values. The von Mises values of the matrix concentrate between the fibers. The matrix damage regions concentrate on where the von-Mises concentrates. With the increase in the loading, the matrix damage also propagates at the microscale (Figure 23a), and the microscale crack is formed at the displacement loading 0.7 mm, as shown in Figure 23b.

**Figure 24:**
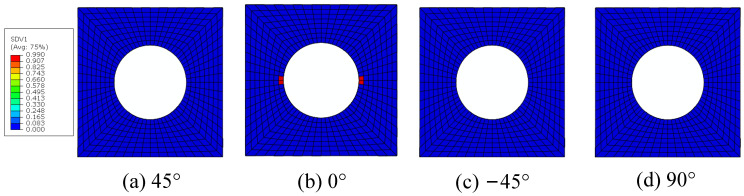
The initiations regions for fiber breakage.

**Figure 25:**
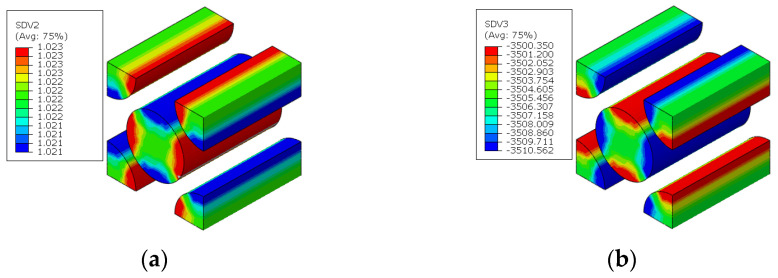
Failure indices and stress states in the fiber for fiber diamond distribution model. (**a**) failure indices; (**b**) compressive stress in the fiber direction.

The fiber breakages initiate around the hole in the 0° ply (Figure 24), and the critical displacement loading for the matrix damage initiation is 0.62 mm. It can also be found that the fiber breaks earlier than the matrix failure, which is consistent with the conclusion obtained from Ref. [16]. For the macroscale element where the fiber breakage initiates first, the microscale failure indices and the stress in the fibers are shown in Figure 25. It can be found that the stress in the fibers along the longitudinal direction are in the compressive stress states. Besides, the stress values in the RVE are almost the same, which contributes to almost the same failure indices in the RVE. It means that all the fiber clusters fail at almost the same time, which demonstrates the validity of adopting the sudden degradation method for fiber breakage (*d_f_*).

**Figure 26:**
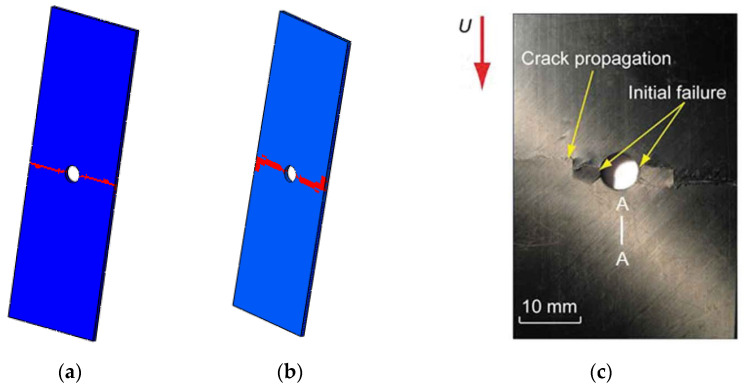
Final failure areas of fiber and matrix. (**a**) fiber failure; (**b**) matrix failure; (**c**) experimental result, reprinted with permission from [16], 2020, © Taylor & Francis.

The failure patterns of the model are shown below. Once the fiber breakage initiates at 0° ply, a large area of matrix cracking and fiber breakage instantaneously takes place along the transverse direction of laminates (Figure 26a,b). Compared with the experimental failure patterns of the open-hole laminates (Figure 26c in Ref. [16]), the similarity between them demonstrates the effectiveness of the modified multiscale method once again.

#### 4.2.2. Effects of Degradation Models

**Figure 27:**
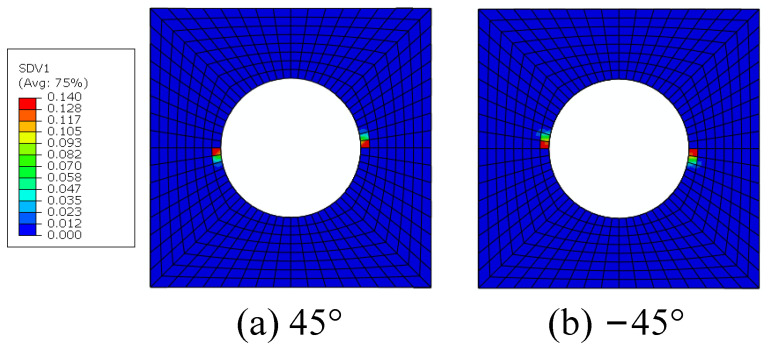
The initiations regions for interface debonding based on FFNN method.

**Figure 28:**
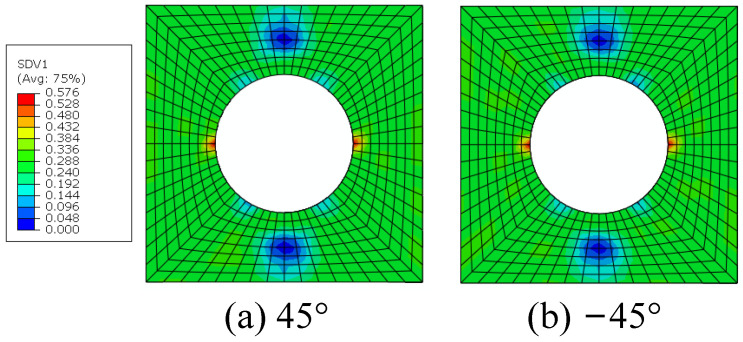
The initiations regions for matrix damage based on FFNN method.

**Figure 29:**
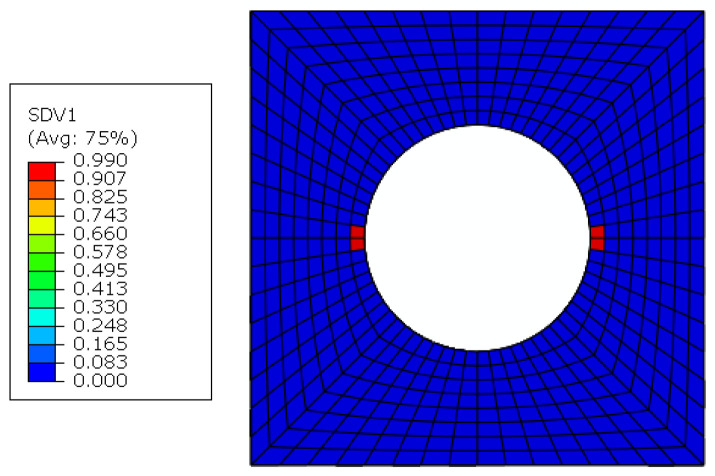
The initiations regions for fiber breakage based on FFNN method.

The interface debonding, matrix damage and fiber breakage initiation patterns of the macroscale model predicted based on the FFNN method are shown in Figure 27, Figure 28 and Figure 29. It can be found that the initiation region is the same with that predicted based on the sudden degradation method. However, under the same external loading condition, the transverse damage value (*d_m,i_*) in Figure 27 predicted based on the FFNN method induced by the interface debonding is larger than 0.11 used in the sudden degradation model. The difference between the damage values should be attributed to the calculation method of the transverse damage value. For the FFNN model, the damage value increases based on the progressive damage processes of the interface, which means that, with the increased failure area of the interface (Figure 20), the transverse damage value becomes larger and larger. However, for the sudden degradation model, the damage vale is set to be 0.11 suddenly once the interface debonding initiates. Thus, under the same displacement loading, a lower macroscale reaction force is obtained for the model based on the FFNN method.

The matrix damage (Figure 28) also initiates at the displacement 0.64 mm. However, when the matrix damage initiates, the transverse damage value is 0.57, which is much smaller than 0.99 set in the sudden degradation method. It means that there is still bearing capability along the transverse direction when the matrix damage initiates. The difference between them should also be attributed to the calculation method of the transverse damage value based on the progressive damage process. With the increase in external loading, the matrix damage area increases at the microscale, as shown in Figure 22, and the transverse damage value increases gradually based on the FFNN model. Thus, it can be concluded that considering the effects of the damage evolution process at the microscale on the transverse damage value based on the FFNN model results in larger predicted compressive strength.

#### 4.2.3. Effects of Fiber Distribution Patterns

**Figure 30:**
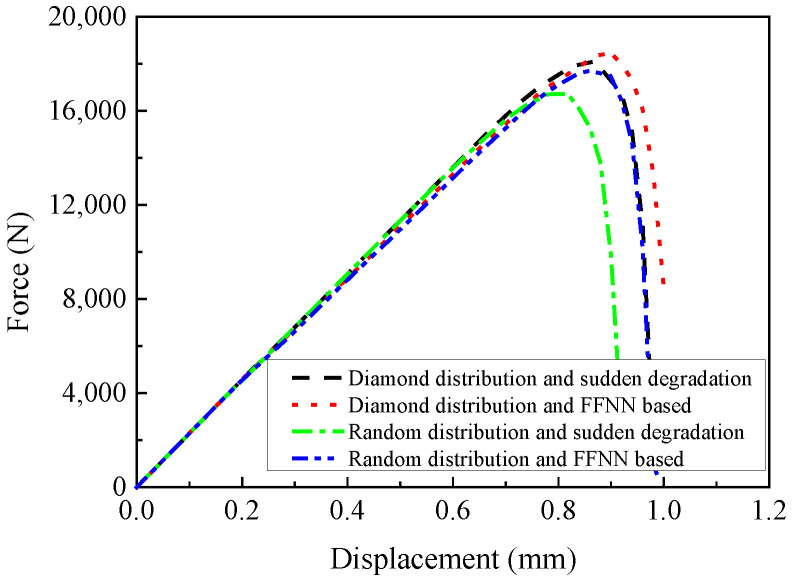
Predicted compressive responses considering different fiber arrangement patterns.

Compared with other studies analyzing compressive behaviors of open-hole laminates [3,4,5,6,7,8,9,10,11], besides the macroscale responses and damage progress, the coupling effects of the interface and fiber random distribution patterns on the predicted results are also revealed in this study. This is because the fiber random distribution pattern and interface can be considered in the modified MMF-based multiscale method. When the fiber random distribution pattern and interface are considered at the microscale, the predicted compressive responses of the open-hole laminates are shown in Figure 30 based on different degradation rules. It can be found that the fiber random distribution pattern decreases the compressive strength dramatically based on the sudden degradation rule (7.2%). Besides, the result predicted based on the FFNN method (17.7 KN) is also larger than that obtained based on the sudden degradation method (16.7 KN), and the increase in percentage is 6.0%. The increase in percentage is larger than that obtained from the fiber diamond distribution model.

The critical displacement loading values of the model with sudden degradation rule are shown in Table 6, and the values represent when each damage mode initiates. It can be found that the interface debonding, matrix damage and fiber breakage initiate much earlier in the model considering the fiber random distribution pattern, especially for the interface debonding and matrix damage. The damage initiation regions at the macroscale are the same as those predicted from the model considering the fiber diamond distribution pattern.

**Figure 31:**
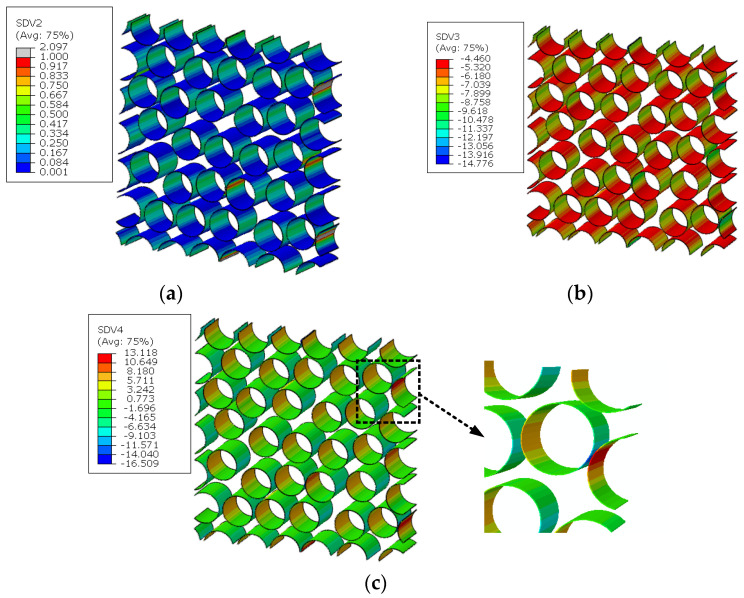
Failure indices and traction force states for the interface. (**a**) failure indices; (**b**) normal traction; (**c**) longitudinal shear traction.

**Figure 32:**
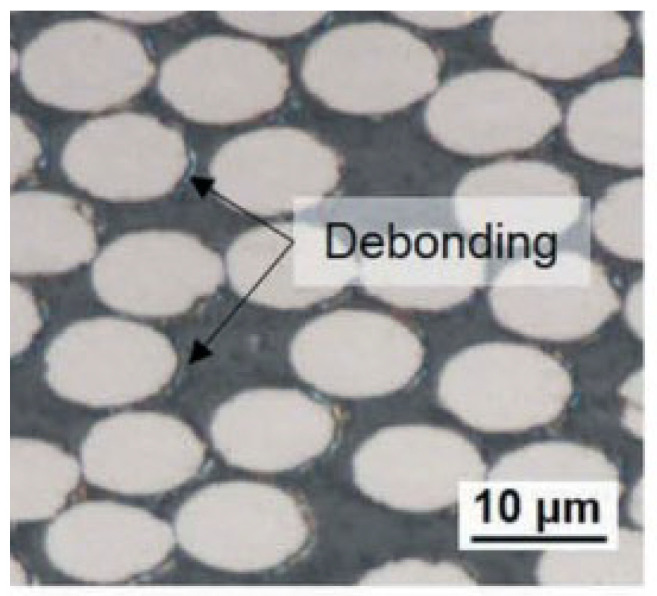
Interface debonding in the 45° ply. Reprinted with permission from [16], 2020, © Taylor & Francis.

**Figure 33:**
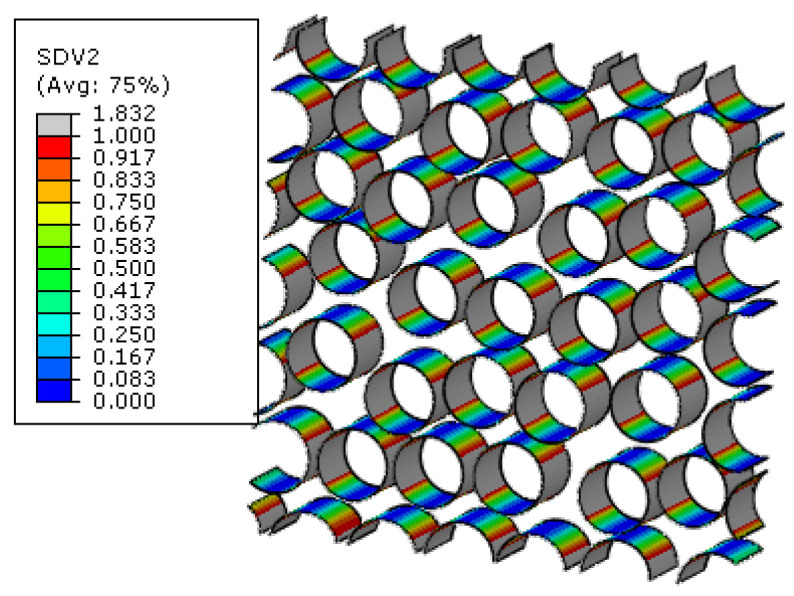
Damage propagation in the interface of fiber random distribution model.

The microscale failure indices and stress distribution patterns of the macroscale elements where each damage model initiates are shown below. For the interface region (Figure 31), the damage and stress concentrate between the fibers with smaller inter-fiber distance. It can be found that the interface debonding also results from the longitudinal shear traction. Due to the much smaller inter-fiber distance, the stress concentration contributes to the much earlier initiation of interface debonding. Compared with the experimental results from Ref. [16] (Figure 32), it can be found that the interface initiates locally due to the fiber random distribution pattern rather than uniformly between the fibers, as shown in Figure 19. This proves the importance of considering the fiber random distribution pattern at the microscale. With the increase in the loading, the damage also propagates to the other interface regions from the initiation region quickly (Figure 33).

**Figure 34:**
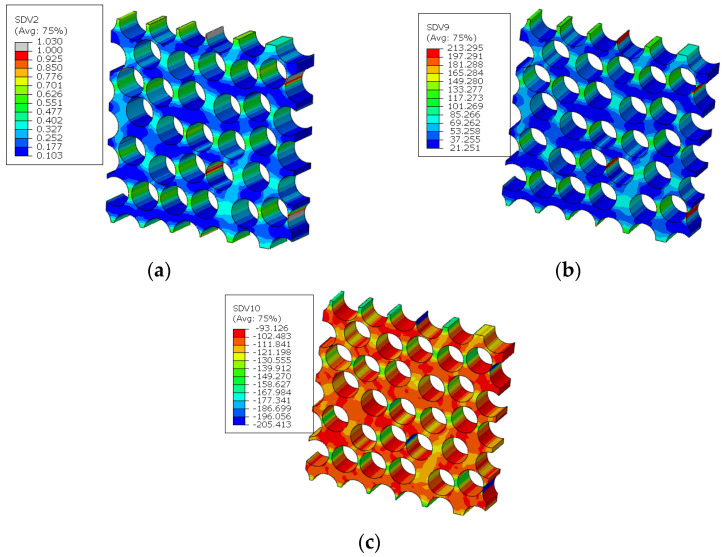
Failure indices and stress states in the matrix of fiber random distribution model. (**a**) failure indices; (**b**) equivalent stress; (**c**) first stress invariant.

**Figure 35:**
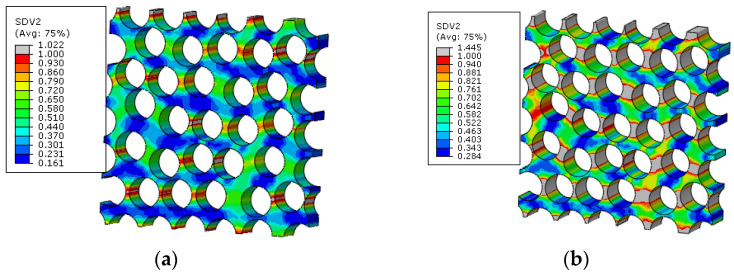
Matrix damage propagation in the matrix of fiber random distribution model. (**a**) at displacement loading 0.4 mm; (**b**) at displacement loading 0.54 mm.

For the matrix, the smaller inter-fiber distance also contributes to the earlier matrix damage initiation (Figure 34) at displacement loading 0.34 mm, which results in the decrease in the predicted compressive strength compared with the result from the model considering the fiber diamond distribution pattern. With the increase in the loading, the area of matrix damage in the RVE increases starting from the matrix regions with less inter-fiber distance (Figure 35a). At last, the microscale crack is formed in the microscale RVE at displacement loading 0.54 mm (Figure 35b). It can be found that the progressive damage process is more complicated and needs larger displacement loading increments for the fiber random distribution model than for the fiber diamond distribution model. Thus, it can be deduced that the larger strength increase percentage for models considering the fiber random distribution pattern should be attributed to the complicated damage process.

**Figure 36:**
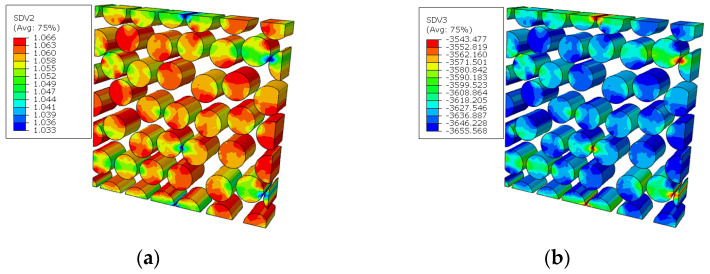
Failure indices and stress states for the fiber. (**a**) failure indices; (**b**) compressive stress in the fiber direction.

The failure indices and stress patterns for the fiber breakage initiation are shown in Figure 36. It can be found that the damage is induced by the compressive stress and the fibers fail at almost the same time, which is the same as the conclusion obtained in the model considering the fiber diamond distribution pattern.

## 5. Conclusions

The micro-mechanics-failure-theory-based multiscale method is modified with the clustering method and the feed-forward neural network. With the clustering method, complex microscale characteristics, including the fiber random distribution pattern and interface, can be considered at the microscale and can be reserved during the multiscale analysis, which is helpful for revealing the failure mechanisms. With the feed-forward neural network, a new degradation method is developed based on the RVE discretized with the clusters. The progressive damage analyses of the open-hole laminates are conducted and the effects of the complex microstructures and the degradation rules are compared in this study. The following conclusions can be drawn below.

1. the predicted results are compared with the published experimental and numerical results first and the agreements between them demonstrate the effectiveness of the modified multiscale method.
2. through comparing the results obtained with and without considering interface debonding, it is found that considering interface results in the premature damage initiation in the laminates.
3. from the results analyzed from the model considering both the fiber random distribution pattern and interface, it is found that, due to the smaller inter-fiber distance, the interface debonding and matrix damage initiate much earlier, which contributes to the decrease in the predicted compressive strength.
4. with the multiscale method developed in this study, both the macroscale and the microscale damage process can be obtained. It is found that, at the microscale, the damage in the fiber random distribution model initiated at a local position rather than uniformly in the fiber diamond distribution model. Besides, the displacement loading increment for the formation of microscale crack in the fiber random distribution model is larger than that in the fiber diamond distribution model.
5. as the progressive damage process of the microscale RVE is considered in the FFNN degradation method for determining the transverse damage value, the predicted strength values based on the FFNN are larger than those obtained based on the sudden degradation method.
6. the difference induced by different degradation rules in the strength values from the models considering the fiber random distribution pattern is much larger than that from the models considering fiber diamond distribution pattern, which should be attributed to the non-uniform damage distribution at the microscale.

## Figures and Tables

**Table 1:** Elastic parameters for the composites and constituents [14,16].

Material Parameters	*E*_11_ (Gpa)	*E*_22_ = *E*_33_ (GPa)	*G*_12_ = *G*_13_ (GPa)	*G*_23_ (GPa)	*v*_12_ = *v*_13_	*v*_23_	*V_f_*
Ply	136	10	4.7	3.2	0.35	0.56	0.56
Fiber	240	42	23	12	0.33	0.71	
Interface	15.9	15.9	5.76	5.76	0.38	0.38	
Matrix	3	3	1.087	1.087	0.38	0.38	

**Table 2:** Strength parameters for the constituents [14,16].

Strength Parameters (MPa)	*T_f_*	*C_f_*	*T_m_*	*C_m_*	*N*	*S*
Values	3710	3430	155	207	18	11.4

**Table 3:** Performance of the trained FFNN for fiber diamond distribution model.

Samples	RMSE (MPa)	*R* ^2^
Training	131.9	0.999
Validation	623.9	0.977
Testing	808.5	0.964

**Table 4:** Performance of the trained FFNN for fiber random distribution model.

Samples	RMSE (MPa)	*R* ^2^
Training	304.7	0.996
Validation	979.1	0.967
Testing	718.8	0.978

**Table 5:** The comparison of the predicted strength values.

Model	Average Experimental Value	In Ref. [16]	In Ref. [14]	With Sudden Degradation Model	With FFNN Model
Strength (KN)	18.6	17.1	18.4	18.0	18.5
Difference percentage (%)	—	−8.065	−1.075	−3.226	−0.538

**Table 6:** Critical displacement values for the initiation of different failure modes.

Models	Interface Debonding	Matrix Damage	Fiber Breakage
Fiber diamond distribution	0.06 mm	0.64 mm	0.62 mm
Fiber random distribution	0.04 mm	0.34 mm	0.58 mm

## Data Availability

The data presented in this study are available on request from the corresponding author.

## Appendix A

The transformation matrix between the global stress values and the traction values is shown in Equation (A1). Then, the traction values in the local coordinates can be obtained with the stress values of interface elements (Equation (A2)), which would be used to judge interface debonding.

(A1) $$T=\left[\begin{array}{cccccc} 0 & \cos^{2}\theta & \sin^{2}\theta & 0 & 0 & 2\cos\theta \sin\theta \\ 0 & 0 & 0 & \cos\theta & \sin\theta & 0\\ 0 & -\cos\theta \sin\theta & \sin\theta \cos\theta & 0 & 0 & \cos^{2}\theta -\sin^{2}\theta \end{array}\right]$$

(A2) $$t=\left[\begin{array}{c} t_{n}\\ t_{x}\\ t_{s} \end{array}\right]=T\sigma$$

where *θ* represents orientation angle between the global coordinates and the local coordinates.

As the traction forces are used for the judgment of interface debonding, the strain–stress amplification factors for the interface are replaced with the strain–traction amplification factors. It is realized with Equation (A3).

(A3) $$t=TA_{\sigma }\bar{\varepsilon }=A_{t}\bar{\varepsilon }$$

## References

[1] Suemasu H., Takahashi H., Ishikawa T. (2006). On failure mechanisms of composite laminates with an open hole subjected to compressive load. Compos. Sci. Technol..

[2] Suemasu H., Naito Y., Gozu K., Aoki Y. (2012). Damage initiation and growth in composite laminates during open hole compression tests. Adv. Compos. Mater..

[3] Zhou S., Sun Y., Chen B., Tay T.E. (2017). Progressive damage simulation of open-hole composite laminates under compression based on different failure criteria. J. Compos. Mater..

[4] Shimizu S., Sato M., Koyanagi J., Suemasu H., Kogo Y. (2021). Numerical simulation of compressive failure of carbon-fiber-reinforced plastic laminates with various hole shapes. Adv. Compos. Mater..

[5] Zhang D., Zheng X., Wu T. (2019). Damage characteristics of open-hole laminated composites subjected to longitudinal loads. Compos. Struct..

[6] Zhao L., Li Y., Zhang J., Zhou L., Hu N. (2018). A novel material degradation model for unidirectional CFRP composites. Compos. Part B. Eng..

[7] Khedkar S., Chinthapenta V., Madhavan M., Ramji M. (2015). Progressive failure analysis of CFRP laminate with interacting holes under compressive loading. J. Compos. Mater..

[8] Zhang H., Shan Y., Guo J., Wen W., Cui H. (2019). Experiments and simulations on the strength of open-hole composite laminates at different temperatures. Mech. Adv. Mater. Struct..

[9] Su Z.C., Tay T.E., Ridha M., Chen B.Y. (2015). Progressive damage modeling of open-hole composite laminates under compression. Compos. Struct..

[10] Zhou S., Zhang J., Sun Y., Tian K. (2019). Experimental and numerical investigation of open hole carbon fiber composite laminates under compression with three different stacking sequences. J. Mater. Res. Technol..

[11] Zhou S., Sun Y., Muhammad R., Chen B., Tay T.E. (2017). Progressive damage simulation of scaling effects on open-hole composite laminates under compression. J. Reinf. Plast. Compos..

[12] Li X., Guan Z., Li Z., Liu L. (2014). A new stress-based multi-scale failure criterion of composites and its validation in open hole tension tests. Chin. J. Aeronaut..

[13] Xu L., Huang Y.C., Zhao C., Ha S.K. (2018). Progressive failure prediction of woven fabric composites using a multi-scale approach. Int. J. Damage Mech..

[14] Li W., Cai H., Li C., Wang K., Fang L. (2014). Progressive failure of laminated composites with a hole under compressive loading based on micro-mechanics. Adv. Compos. Mater..

[15] Liu Z., Guan Z., Tan R., Xu J. (2019). Analysis of open-hole compressive CFRP laminates at various temperatures based on a multiscale strategy. Appl. Compos. Mater..

[16] Lou X., Han X., Cai H. (2020). A high-efficient model for interface debonding analysis of carbon fiber-reinforced polymer composite. Compos. Interfaces.

[17] Wang M. (2021). A Multiscale Method Across Three Length Scales for Progressive Damage Analysis of Plain Woven Composites. Appl. Compos. Mater..

[18] Wang M., Zhang P., Fei Q., Guo F. (2020). Modified micro-mechanics based multiscale model for progressive failure prediction of 2D twill woven composites. Chin. J. Aeronaut..

[19] Gu Y., Li M., Wang J., Zhang Z. (2010). Characterization of the interphase in carbon fiber/polymer composites using a nanoscale dynamic mechanical imaging technique. Carbon.

[20] Wang B., Fang G., Liu S., Liang J. (2019). Effect of heterogeneous interphase on the mechanical properties of unidirectional fiber composites studied by FFT-based method. Compos. Struct..

[21] Elnekhaily S.A., Talreja R. (2018). Damage initiation in unidirectional fiber composites with different degrees of nonuniform fiber distribution. Compos. Sci. Technol..

[22] Borkowski L.B., Liu K.C., Chattopadhyay A. (2013). From ordered to disordered: The effect of microstructure on composite mechanical performance. Comput. Mater. Contin..

[23] Wang M., Zhang P., Fei Q., Guo F. (2019). Computational evaluation of the effects of void on the transverse tensile strengths of unidirectional composites considering thermal residual stress. Compos. Struct..

[24] Bheemreddy V., Chandrashekhara K., Dharani L.R., Hilmas G.E. (2016). Computational study of micromechanical damage behavior in continuous fiber-reinforced ceramic composites. J. Mater. Sci..

[25] Wang Z., Wang X., Zhang J., Liang W., Zhou L. (2011). Automatic generation of random distribution of fibers in long-fiber-reinforced composites and mesomechanical simulation. Mater. Des..

[26] Jin K.K., Huang Y., Lee Y.H., Ha S.K. (2008). Distribution of micro stresses and interfacial tractions in unidirectional composites. J. Compos. Mater..

[27] Liu Z., Bessa M.A., Liu W.K. (2016). Self-consistent clustering analysis: An efficient multi-scale scheme for inelastic heterogeneous materials. Comput. Methods Appl. Mech. Eng..

[28] Liu Z., Fleming M., Liu W.K. (2018). Microstructural material database for self-consistent clustering analysis of elastoplastic strain softening materials. Comput. Methods Appl. Mech. Eng..

[29] Han X., Gao J., Fleming M., Xu C., Xie W., Meng S., Liu W.K. (2020). Efficient multiscale modeling for woven composites based on self-consistent clustering analysis. Comput. Methods Appl. Mech. Eng..

[30] Yan S., Zou X., Ilkhani M., Jones A. (2020). An efficient multiscale surrogate modelling framework for composite materials considering progressive damage based on artificial neural networks. Compos. Part B. Eng..

[31] Zhang Z., Friedrich K., Velten K. (2002). Prediction on tribological properties of short fibre composites using artificial neural networks. Wear.

[32] Zhang Z., Friedrich K. (2003). Artificial neural networks applied to polymer composites: A review. Compos. Sci. Technol..

[33] Lemaitre J. (2012). A Course on Damage Mechanics.

[34] Cybenko G. (1989). Approximation by superpositions of a sigmoidal function. Math. Control. Signals Syst..

[35] Hornik K., Stinchcombe M., White H. (1989). Multilayer feedforward networks are universal approximators. Neural Netw..

[36] Zhao Z., Wang Z., Yuan J., Ma J., He Z., Xu Y., Shen X., Zhu L. (2021). Development of a novel feedforward neural network model based on controllable parameters for predicting effluent total nitrogen. Engineering.

[37] Li W., Cai H., Li C. (2014). Static compressive strength prediction of open-hole structure based on non-linear shear behavior and micro-mechanics. Mech. Time-Depend. Mater..

